# Rhino‐Orbital‐Cerebral Mucormycosis in a Patient With Chronic Hepatitis B and D Coinfection: A Rare Case Report

**DOI:** 10.1002/ccr3.71707

**Published:** 2025-12-17

**Authors:** Fazeela Bibi, Muhammad Hamza, Osvani Leyva Matos, Manal Mohsin, Alizah fareed, Fareena Ambreen, Khalil El Abdi, Said Hamid Sadat

**Affiliations:** ^1^ Department of Medicine Jinnah Medical and Dental College Karachi Pakistan; ^2^ Department of Medicine Saidu Medical College Swat Pakistan; ^3^ American University of the Caribbean School of Medicine Caribbean USA; ^4^ Department of Medicine M. Islam Medical & Dental College Gujranwala Pakistan; ^5^ Department of Medicine Sir Syed College of Medical Sciences Karachi Pakistan; ^6^ Department of Medicine Khyber Girls Medical College Peshawar Pakistan; ^7^ Faculty of Medicine and Pharmacy of Rabat Mohammed V University Rabat Morocco; ^8^ Kabul University of Medical Science Abu Ali ibn Sina Kabul Afghanistan

**Keywords:** hepatitis B virus (HBV), hepatitis D virus (HDV), immunocompromised host, liposomal amphotericin B, mucormycosis, rhino‐orbital‐cerebral mucormycosis (ROCM)

## Abstract

Rhino‐orbital‐cerebral mucormycosis (ROCM) is a rare but highly lethal fungal infection that typically occurs in patients with classical immunosuppressive states. This report presents a case of ROCM in a 27‐year‐old male whose sole systemic risk factor was chronic Hepatitis B (HBV) and Hepatitis D (HDV) coinfection. The patient presented with advanced disease, including nasal necrosis and progressive periorbital swelling. Advanced imaging revealed extensive intraorbital and intracranial invasion with significant mass effect, a finding that rendered the standard‐of‐care surgical debridement prohibitively high risk. A definitive diagnosis was established via histopathology. Consequently, a deliberate decision was made to proceed with exclusive medical management. The patient was treated successfully with high‐dose intravenous liposomal amphotericin B monotherapy, resulting in a favorable clinical outcome. This case establishes chronic viral hepatitis as a significant non‐classical risk factor for ROCM and provides critical evidence that aggressive medical monotherapy can serve as a definitive, life‐saving treatment when the standard bimodal surgical approach is contraindicated.

**Key Clinical Message**

- Chronic hepatitis is a standalone risk factor for mucormycosis. When surgery is off the table, aggressive liposomal amphotericin B monotherapy is not just an alternative—it can be a life‐saving definitive treatment.

## 1 Introduction

Among invasive fungal infections, mucormycosis is the third most common cause [1]. This life‐threatening condition results from infections caused by Mucorales fungi and is associated with significant mortality rates in hospitalized patients [2]. Although these infections are rare, a rising number of instances are being reported in immunocompromised individuals. Major risk factors for mucormycosis include poorly controlled diabetes, prolonged high‐dose glucocorticoid use, neutropenia, organ transplantation, iron overload, and, more recently, COVID‐19 infection [3, 4]. Mucorales infections can manifest in different ways, such as cutaneous and soft tissue infections, rhino‐orbito‐cerebral disease, pulmonary or gastrointestinal involvement, disseminated infection, and other rare presentations. Diagnosis relies on detecting distinctive, broad hyphae in tissue specimens or isolating the fungus through mycological culture. Delayed treatment significantly raises mortality [5, 6, 7]. However, the clinical presentation of ROCM exists on a spectrum. The classic fulminant form is characterized by rapid progression over several days, while chronic invasive forms can evolve over weeks to months, a distinction with critical implications for management. Chronic HBV infection may lead to varying degrees of liver dysfunction and immune dysregulation, potentially increasing susceptibility to opportunistic pathogens [8, 9]. In this report, we present a rare and clinically significant case of mucormycosis in a patient with chronic hepatitis B. This case is notable for its protracted clinical history, suggestive of a chronic invasive process that culminated in an acute exacerbation. Therefore, we aim to explore chronic viral hepatitis as a predisposing factor for this unusual presentation and to demonstrate that in cases where extensive intracranial disease contraindicates surgery, medical monotherapy can be a viable life‐saving strategy.

## 2 Case Presentation

A 27‐year‐old male with a known history of chronic Hepatitis B Virus (HBV) infection, complicated by a recent Hepatitis D Virus (HDV) coinfection, presented to our tertiary care center. The patient's clinical course, summarized in Table 1, was highly suggestive of a chronic, invasive fungal process that had transitioned into an acute, fulminant state.

**TABLE 1:** Clinical timeframe of the history of present illness.

Timeframe	Clinical events and milestones
~4 Months prior to admission	Onset of slowly progressive left periorbital swelling extending to the nasal bridge.
1 Month prior to admission	Development of intermittent epistaxis (nasal bleeding).
2 Weeks prior to admission	Acute clinical deterioration marked by the onset of a severe, unremitting headache.
Day of admission	Presentation to tertiary care center. Physical exam reveals palatal necrosis. Diagnosis of ROCM is confirmed by histopathology from a nasal tissue biopsy.
Post‐admission	Advanced imaging (MRI and CT) confirms extensive rhino‐orbital‐cerebral disease with mass effect.

### 2.1 History of Present Illness

The patient's illness began insidiously approximately 4 months prior to presentation, with the onset of a slowly progressive, left‐sided periorbital swelling that extended to the nasal bridge. One month prior to admission, he developed intermittent epistaxis (nasal bleeding). The clinical course acutely worsened in the 2 weeks preceding admission, culminating in the development of a severe, unremitting headache that prompted him to seek emergency care. His past medical history was significant for poor compliance with his prescribed antiviral regimen for HBV. His social history included a multi‐year pattern of chronic naswar (smokeless tobacco) use. Critically, no traditional risk factors for mucormycosis, such as diabetes mellitus, recent glucocorticoid use, hematologic malignancy, or other known immunodeficiencies, were identified.

### 2.2 Clinical Examination and Diagnostic Findings

On physical examination at the time of admission, the patient exhibited marked, tender edema of the left periorbital region, extending across the nasal bridge. Intraoral examination was remarkable for a large, black, necrotic eschar covering a significant portion of the hard palate, a hallmark sign of advanced angioinvasive fungal disease. Given the high suspicion for rhino‐orbital‐cerebral mucormycosis (ROCM), an urgent consultation with the otolaryngology service was obtained. Subsequent nasal endoscopy and tissue biopsy were performed. Histopathological analysis was conclusive, revealing broad, non‐septate, ribbon‐like fungal hyphae with evidence of angioinvasion, confirming the diagnosis of mucormycosis. Laboratory investigations confirmed active HBV/HDV coinfection but showed hematological and biochemical parameters within normal limits, notably an absence of neutropenia.

### 2.3 Advanced Imaging

**FIGURE 1:**
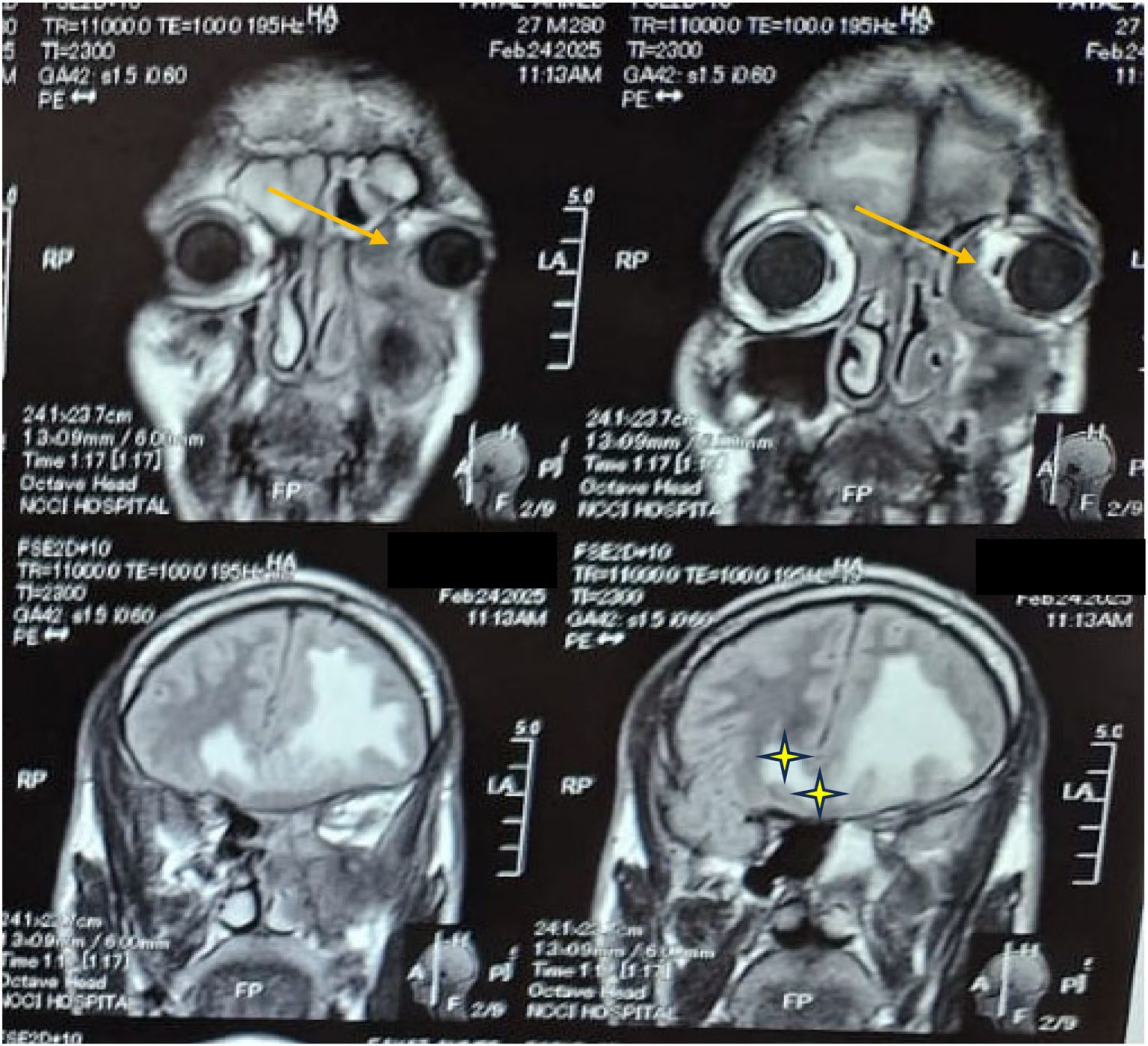
Magnetic resonance imaging (MRI) revealing midline shift to the right (asterisk) and lateral displacement of the left globe.

**FIGURE 2:**
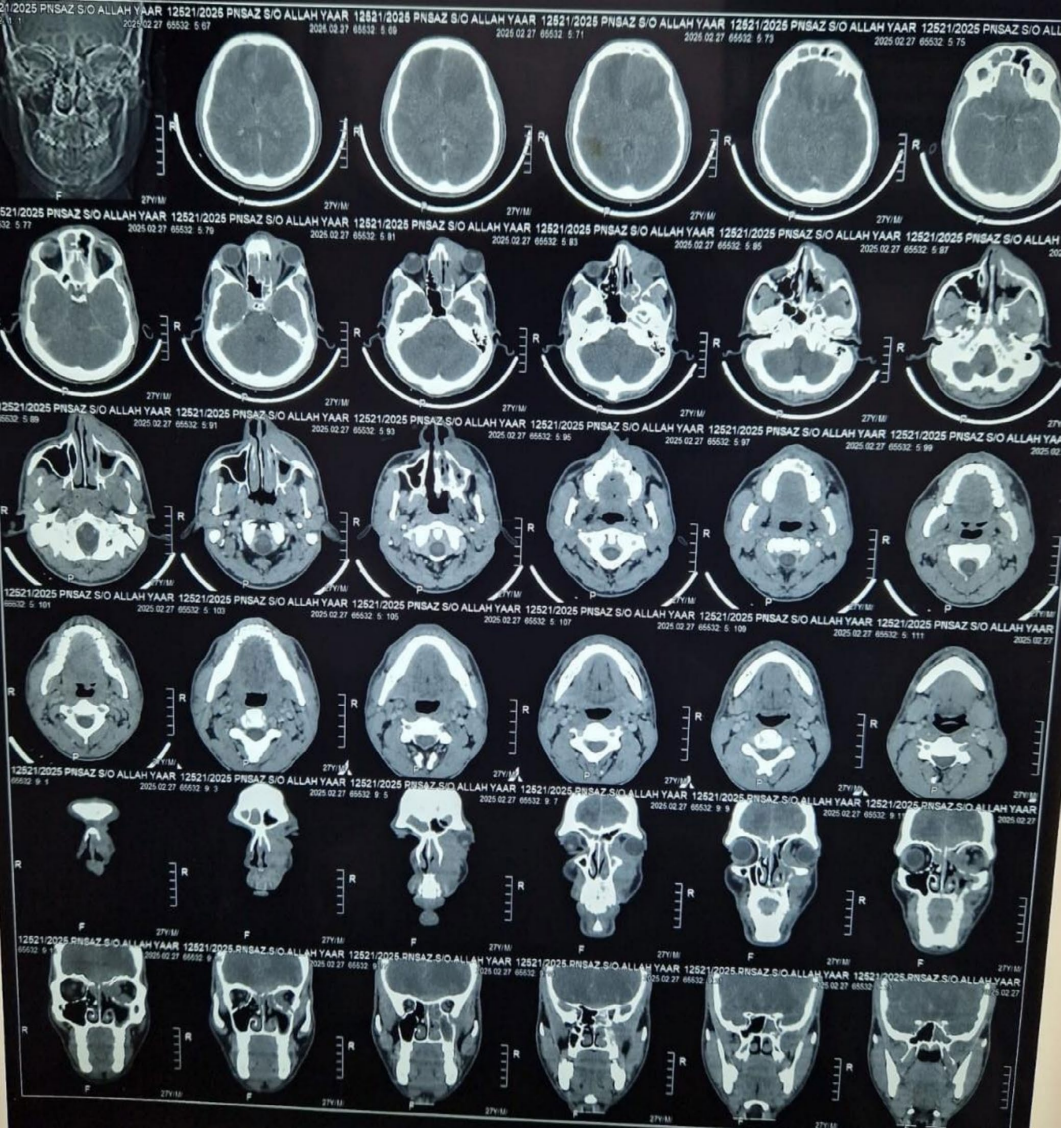
Computed tomography (CT) demonstrating sinonasal and orbital involvement. Axial, non‐contrast computed tomography scan of the paranasal sinuses. The image reveals extensive soft‐tissue density opacification of the left ethmoid and sphenoid sinuses. There is clear evidence of bony erosion, particularly involving the lamina papyracea (the medial wall of the orbit, indicated by the white arrow), signifying invasive disease with extension into the left orbit. Associated inflammatory changes are noted in the adjacent preseptal soft tissues.

Following the definitive diagnosis, urgent advanced imaging was conducted to stage the extent of the disease. Magnetic resonance imaging (MRI) (Figure 1) of the brain and orbits revealed extensive intracranial and intraorbital disease, resulting in lateral displacement of the left globe, marked cerebral edema, and a significant midline shift to the right. To assess osseous involvement, a computed tomography (CT) (Figure 2) scan of the paranasal sinuses was performed. The CT demonstrated erosion of the medial orbital wall and documented the spread of infection into the nasal cavity, the infra‐temporal fossa, and the left orbit, where it caused compression of the medial rectus muscle.

### 2.4 Therapeutic Management and Clinical Outcome

The patient was promptly initiated on a standard institutional protocol of intravenous liposomal amphotericin B. In light of the extensive intracranial involvement identified on imaging, surgical debridement was considered high risk and was deferred in favor of exclusive medical management.

The patient was monitored closely and demonstrated early clinical improvement, particularly in the resolution of his orbital symptoms. His antifungal therapy was well tolerated, with no evidence of significant renal or electrolyte complications. Following a prolonged hospital course, he was discharged in a stable condition with arrangements made for close multidisciplinary follow‐up to monitor both his fungal infection and his chronic viral hepatitis.

Follow‐up magnetic resonance imaging (MRI) performed 3 months after initiation of antifungal therapy demonstrated significant regression of intracranial lesions, with marked reduction in cerebral edema and resolution of midline shift. These radiological improvements correlated with the patient's sustained clinical recovery, confirming the effectiveness of exclusive liposomal amphotericin B monotherapy in this high‐risk case.

## 3 Discussion

This case is instructive on two primary fronts: first, it identifies chronic Hepatitis B and D coinfection as a significant, standalone risk factor for the development of rhino‐orbital‐cerebral mucormycosis (ROCM); and second, it demonstrates the potential for high‐dose liposomal amphotericin B monotherapy to serve as a definitive, life‐saving treatment when extensive intracranial disease contraindicates surgical intervention.

The presentation of ROCM in this patient challenges the conventional hierarchy of risk factors, which are typically dominated by poorly controlled diabetes mellitus, hematologic malignancies, and prolonged glucocorticoid use [10]. While the recent COVID‐19 pandemic has also been identified as a major predisposing condition [11], the role of chronic viral hepatitis has remained largely uncharacterized. We propose that the patient's susceptibility was driven by virus‐induced immune dysregulation, a well‐documented phenomenon in chronic HBV/HDV infection characterized by T‐cell exhaustion and diminished cytokine responses [12]. This underlying immunodeficiency likely created a permissive state for this aggressive opportunistic infection, even in the absence of neutropenia or other classical immunosuppressive conditions.

The diagnosis was confirmed via histopathology, which revealed the characteristic broad, non‐septate, angioinvasive hyphae of mucorales [13]. Advanced imaging was critical in staging the disease and guiding the therapeutic strategy. While CT is superior for delineating bony erosion, MRI was indispensable in visualizing the extensive intracranial and soft‐tissue spread, including the mass effect and midline shift that defined the patient's high‐risk status [14]. It was these MRI findings that created a therapeutic crossroads. The standard of care for ROCM is a bimodal approach combining systemic antifungal therapy with aggressive, early surgical debridement of all necrotic tissue [15]. However, the profound intracranial involvement rendered any surgical attempt prohibitively dangerous, with an unacceptable risk of catastrophic neurological outcomes.

Faced with this clinical dilemma, the decision was made to proceed with exclusive medical management using intravenous liposomal amphotericin [16]. The patient's favorable clinical response (clinical improvement and follow‐up imaging) and eventual discharge in a stable condition are therefore highly significant. This outcome contributes to an emerging body of evidence suggesting that in select patients for whom surgery is contraindicated, aggressive antifungal monotherapy can be a viable and life‐saving alternative to the standard bimodal approach.

We acknowledge the potential confounding role of the patient's chronic use of naswar (smokeless tobacco), which may have caused local mucosal damage and created a portal of entry for the fungal spores. However, the progression from a localized inoculation to a deeply angioinvasive, intracranial disease strongly indicates that a systemic immunodeficiency—most plausibly from the uncontrolled viral hepatitis—was the primary driver of this fulminant infection. The principal limitation of this report is that, as a single case study, it cannot establish causality between HBV/HDV and ROCM susceptibility. Furthermore, the hypothesis of virus‐induced immunodeficiency is based on established literature rather than direct immunological assays from the patient. Despite these limitations, this case underscores the need for clinicians to maintain a high index of suspicion for ROCM in patients with chronic viral hepatitis who present with necrotic facial or orbital lesions, even without traditional risk factors.

## 4 Conclusion

This case establishes chronic HBV/HDV coinfection as a significant, non‐classical risk factor for rhino‐orbital‐cerebral mucormycosis and confirms the viability of aggressive liposomal amphotericin B monotherapy as a definitive treatment when extensive intracranial disease contraindicates surgical debridement. Consequently, a high index of suspicion for ROCM is warranted in patients with chronic viral hepatitis who present with compatible sinonasal or orbital pathology, regardless of classical risk factors. This report underscores the urgent need for further investigation into the immunological pathways linking chronic viral hepatitis to fungal susceptibility, which is essential for refining risk stratification and optimizing therapeutic strategies in this emerging at‐risk population.

## Author Contributions

**Fazeela Bibi:** project administration, supervision, validation, visualization, writing – original draft, writing – review and editing. **Muhammad Hamza:** methodology, project administration, supervision, validation, visualization, writing – original draft, writing – review and editing. **Osvani Leyva Matos:** data curation, formal analysis, investigation, methodology. **Manal Mohsin:** data curation, formal analysis. **Alizah fareed:** investigation, methodology, project administration. **Fareena Ambreen:** methodology, project administration, resources. **Khalil El Abdi:** data curation, formal analysis, investigation, methodology, project administration, resources, supervision, validation, visualization, writing – original draft, writing – review and editing. **Said Hamid Sadat:** resources, validation, visualization.

## Funding

The authors have nothing to report.

## Ethics Statement

Ethics approval and consent to participate were obtained from the individual participant in the study. The participant has consented for the submission of the case report to the journal. Patient signed informed consent regarding publishing his data and photographs.

## Conflicts of Interest

The authors declare no conflicts of interest.

## Data Availability

The data was taken from a patient who presented to our hospital, all data and references are publicly available on databases such as Pub‐med and Google Scholar.
